# Global Nav1.7 Knockout Mice Recapitulate the Phenotype of Human Congenital Indifference to Pain

**DOI:** 10.1371/journal.pone.0105895

**Published:** 2014-09-04

**Authors:** Jacinthe Gingras, Sarah Smith, David J. Matson, Danielle Johnson, Kim Nye, Lauren Couture, Elma Feric, Ruoyuan Yin, Bryan D. Moyer, Matthew L. Peterson, James B. Rottman, Rudolph J. Beiler, Annika B. Malmberg, Stefan I. McDonough

**Affiliations:** 1 Department of Neuroscience, Amgen Inc., Cambridge, Massachusetts, United States of America; 2 Department of Pharmaceutics Research & Development, Amgen Inc., Cambridge, Massachusetts, United States of America; 3 Department of Pathology, Amgen Inc., Cambridge, Massachusetts, United States of America; 4 Department of Comparative Animal Research, Amgen Inc., Cambridge, Massachusetts, United States of America; University of South California, United States of America

## Abstract

Clinical genetic studies have shown that loss of Nav1.7 function leads to the complete loss of acute pain perception. The global deletion is reported lethal in mice, however, and studies of mice with promoter-specific deletions of Nav1.7 have suggested that the role of Nav1.7 in pain transduction depends on the precise form of pain. We developed genetic and animal husbandry strategies that overcame the neonatal-lethal phenotype and enabled construction of a global Nav1.7 knockout mouse. Knockouts were anatomically normal, reached adulthood, and had phenotype wholly analogous to human congenital indifference to pain (CIP): compared to littermates, knockouts showed no defects in mechanical sensitivity or overall movement yet were completely insensitive to painful tactile, thermal, and chemical stimuli and were anosmic. Knockouts also showed no painful behaviors resulting from peripheral injection of nonselective sodium channel activators, did not develop complete Freund’s adjuvant-induced thermal hyperalgesia, and were insensitive to intra-dermal histamine injection. Tetrodotoxin-sensitive sodium current recorded from cell bodies of isolated sensory neurons and the mechanically-evoked spiking of C-fibers in a skin-nerve preparation each were reduced but not eliminated in tissue from knockouts compared to littermates. Results support a role for Nav1.7 that is conserved between rodents and humans and suggest several possibly translatable biomarkers for the study of Nav1.7-targeted therapeutics. Results further suggest that Nav1.7 may retain its key role in persistent as well as acute forms of pain.

## Introduction

Clinical genetic studies from several groups have shown that loss of function of the Nav1.7 (SCN9A) voltage-gated sodium ion channel leads to complete inability to perceive pain (congenital indifference to pain or CIP) [1]–[3], whereas Nav1.7 gain of function alleles cause or contribute to chronic spontaneous pain [4]–[7]. Mechanistically, Nav1.7 governs the excitability of peripheral nociceptive sensory neurons and the transmission of noxious nociceptive signals into the spinal cord [8]–[10], possibly by facilitating action potential propagation across fine neuronal branch points [11]–[14]. Evidence also is mounting that Nav1.7 plays a major role in transducing pain in acquired clinical neuropathic pain syndromes [15]–[18].

Although human CIP patients survive to adulthood, global Nav1.7 deletion has been reported neonatal lethal in mice [19],[20], and behavioral tests of mice with tissue-specific Nav1.7 deletions have been interpreted to infer quite complex roles for Nav1.7 in different forms of experimental pain [19],[21],[22]. This is in contrast to human CIP, in which patients are insensitive to all painful stimuli, although the experimental pain protocols used to test mice are not necessarily analogous to the experience of CIP patients. Likewise, studies of mice with deletion of non-Nav1.7 sodium channels suggest that Nav1.7 is not the only sodium channel that governs the excitability of peripheral pain pathways [22]–[25]. One explanation is the expanded distribution of Nav1.7 reported in rodent compared to primate brain, which suggests species-specific functions [3]. Alternatively, Nav1.7 could have a crucial function preserved in CIP alleles but lost in the mouse knockout construct. Given the key role of Nav1.7 in human pain, it has been somewhat surprising to find a more nuanced role for the gene in mice, since the translation of pain pharmacology from rodent models to human clinical use is generally quite good [26] and suggests evolutionary conservation of basic pain mechanisms. Despite the great interest in developing selective Nav1.7 antagonists as novel analgesics, it is not clear what preclinical models of pain necessarily reflect Nav1.7 activity and so might be used to prioritize compounds and select doses for clinical trials.

To help address the physiological role of Nav1.7 and to aid the development of new therapeutics, we have made a global knockout of Nav1.7 and profiled the knockouts in a variety of behavioral assays, including a novel assay reflecting direct pharmacological activation of sodium channels. Mice missing Nav1.7 were insensitive to thermal, tactile, and chemical pain, to the induction of chronic pain by CFA treatment, to nonselective sodium channel activators, to histamine challenge, and to olfactory stimulus, but other non-painful behavior as observed was normal. Electrophysiological recordings show that some fast TTX-sensitive current remained in the cell bodies of sensory neurons from knockouts, and that spiking in C-fibers was reduced but still present. Characterization of these mice suggests several possibly translatable behavioral biomarkers that could reflect target occupancy of Nav1.7 by an experimental therapeutic. We speculate that Nav1.7 is an evolutionarily conserved molecular control point for the transmission of high-threshold noxious stimuli into the spinal cord.

## Results

### Generation of Nav1.7 global knockout mice

For this mouse model, we began with the commercially available mice generated by Deltagen: B6.129P2-Scn9a^tm10gen^/J Deltagen. These mice carry one intact Nav1.7 allele and one disrupted by an insertion of bacterial *lacZ* and were made congenic. In our hands, when C57BL/6 mice heterozygous for Nav1.7 (+/−; HET) were intercrossed, no viable homozygous Nav1.7 knockout (−/−; KO) mice were obtained, confirming previous reports of neonatal lethality [19],[20]. C57BL/6 mice tend to be poor nurturers. We speculated that lethality was not due to absence of Nav1.7 itself, but that better maternal care in addition to modifications to the genetic background to increase vigor might help the survival of this otherwise inbred strain. Accordingly, C57BL/6-Nav1.7 HET mice were out-crossed to the CD1 outbred strain, as outbred mice in general are more robust than inbred. The resulting Nav1.7 HET hybrids (approximately 50% C57BL/6 and 50% CD1) from this outcrossing were bred to produce Nav1.7 KO. In parallel, C57BL/6 Nav1.7 HET mice were back-crossed to BALB/c mice, generally known to provide better maternal care and a more favorable background for survival of mutants. Nav1.7 hybrid HETs from this BALB/c cross were 1) further back-crossed onto BALB/c mice to generate congenic BALB/c-Nav1.7 HET mice, and 2) bred as hybrids to generate Nav1.7 KO animals. Both filial and non-filial breeding schemes were tested for all variants generated. Given the acknowledged differences in pain sensitivity among inbred strains of mice [27], we also hoped to assess the effects of Nav1.7 deletion on multiple strain backgrounds.

**Figure 1:**
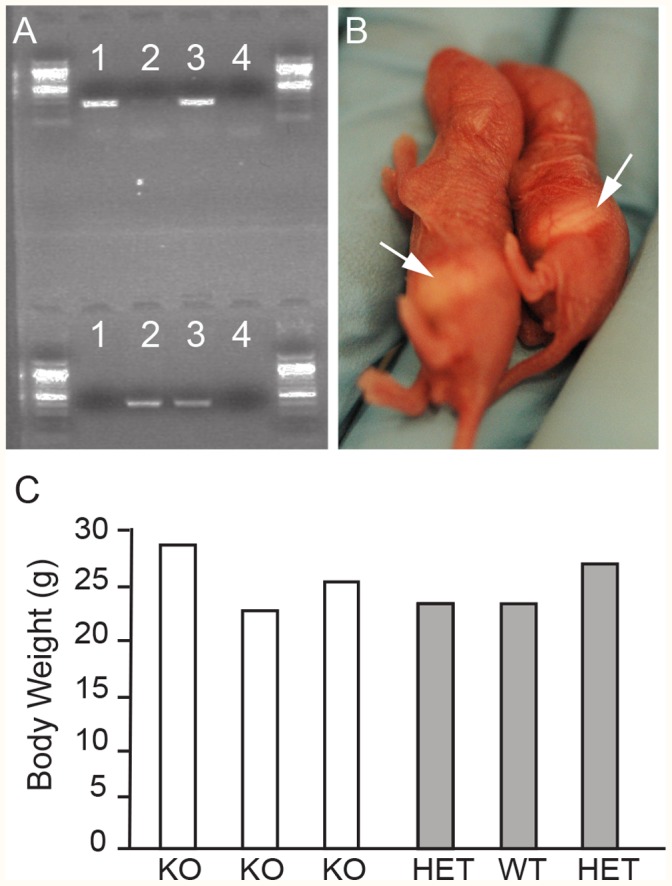
Genotyping and phenotyping of global Nav1.7 knockout animals. **A.** Example of endogenous (top - 267 bp) and targeted (bottom - 389 bp) PCR products obtained from DNA isolated from Nav1.7 WT (lane 1), KO (lane 2) and HET (lane 3) animals, while the last lane serves as a negative control (no DNA). All PCRs were run using a 1 kb PLUS DNA ladder. **B.** Photos of postnatal day 2 littermates showing their capability in feeding naturally by the presence of milk spots in control (see arrow left) and KO (see arrow right) and slight size difference in young animals. **C.** Body weight (grams) comparison between six female littermates at nine weeks of age that represented all three genotype classes illustrating the absence of size difference in adult animals.

The effects of Nav1.7 KO on survival of the CD1 strain were examined first. Global CD1-Nav1.7 KO pups, with genotype verified by PCR (Figure 1A), were indeed born from the hybrid parents and could survive independently up to postnatal day seven (P7; see milk spots in Figure 1B). For the most part, the CD1 Nav1.7 KO neonates were smaller than littermates (Figure 1B). A number of steps were taken to increase mouse survival. CD1-Nav1.7 KO mice were fed by hand two to three times a day using an in-house modified artificial mouse milk formula that mimicked the composition of mouse milk [28],[29]. In addition, neonates showing signs of dehydration also received supplementary dextrose injections subcutaneously up to three times a day. Litters that contained CD1-Nav1.7 potential KO mice were culled to contain enough homozygous wild type (WT) or HET mice to ensure sufficient lactation and to keep the neonates warm, but not so many WT/HET mice as to trample or out-compete the KO mice for resources. Supplemental feeding was critical during the first two postnatal weeks, but once the neonates’ teeth erupted at approximately P14 they were able to feed on moist food and supplements placed at the bottom of the cage. Using such methods, and with continuing animal husbandry and care, mice were obtained that grew to adulthood. With experience with these methods, KO mice were obtained consistently, albeit at a success rate lower than the expected Mendelian ratio. Parallel breeding and nurturing of the hybrid BALB/c strain also produced adult BALB/c Nav1.7 KO mice, but breeding and nurturing of the congenic BALB/c Nav1.7 did not.

A total of 130 adult knockouts were produced from the CD1 and BALB/c background hybrid strains from a total of ∼2,450 neonatal knockouts, showing high hurdles to survival even with these more vigorous hybrid background strains and with intensive husbandry. With optimization of procedures, we have been able to achieve a ∼20% survival rate in the expected knockout population. Most loss occurred prior to P14. Past the neonatal phase, KOs gained weight at the same rate as HET and WT animals (data not shown) and adults reached similar weight range as control littermates (Figure 1C), suggesting that the lower neonatal weight was most likely due to low calorie intake and not to any direct metabolic function of Nav1.7. In early stages of production where F1 and F2 were bred (thus affecting the hybrid contribution of both backgrounds), resulting mice often showed an increase in dermatitis, a common trait of C57Bl/6 strains. Some Nav1.7 KO mice, mainly male, also presented with urinary issues such as prolapses and blockage and required manual expression of their bladders. These and other health problems were in good measure prevented in subsequent generations by limiting the Nav1.7 KO production to breeding of F1 CD1/BALB/c Nav1.7 (50∶50) HETs only, and by not breeding F2 generation or beyond. Male and female Nav1.7 KO mice each were fertile.

### Anatomy and behavior of Nav1.7 global knockout mice

**Figure 2:**
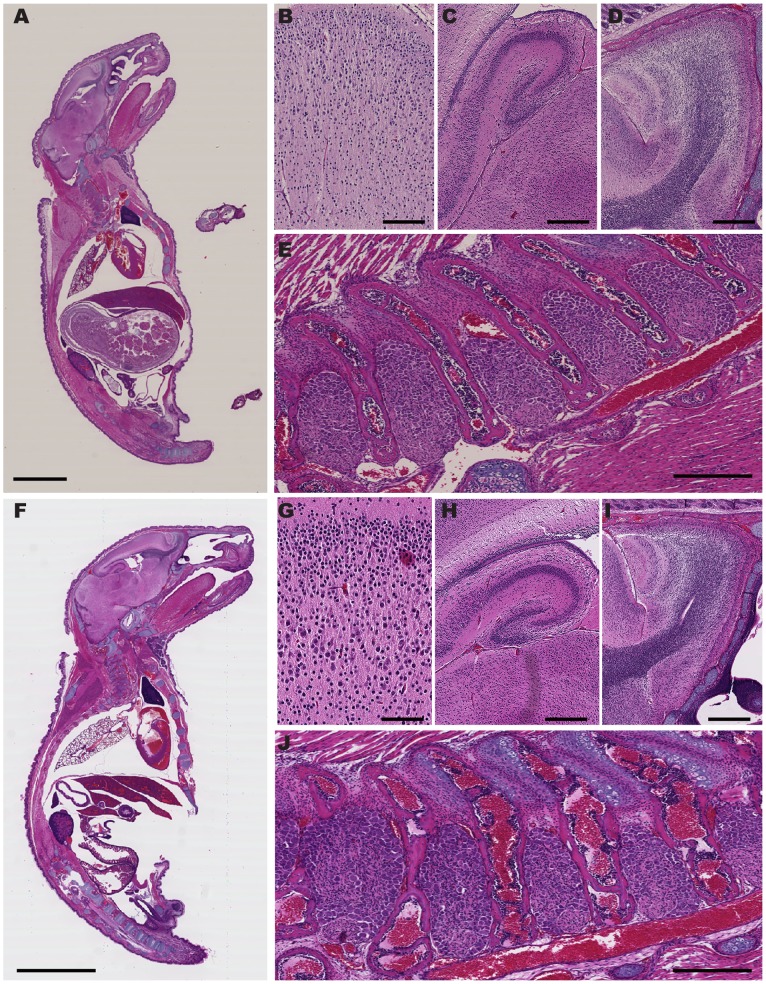
Normal anatomy of Nav1.7 KOs. Postnatal day 4 Nav1.7 WT (A to E) and KO (F to J) neonates stained with hematoxylin and eosin (H&E). **Wild type:** **A.** Sagittal section of the entire pup. **B** through **E** show magnifications of various regions of the central and peripheral nervous systems: **Knock Out:** **F.** Sagittal section of the entire pup. **G** through **J** show magnifications of various regions of the central and peripheral nervous systems. (**B, G**) cortex; (**C, H**) hippocampus; (**D, I**) olfactory bulb; (**E, J**) dorsal root ganglia. (Scale bars: **A, F** = 5 mm; **B, G** = 100 µm; **C, H** and **D, I** = 400 µm; **E, J** = 300 µm).

In overall behavior and anatomy, Nav1.7 KOs from both CD1 and BALB/c strains appeared normal. To examine organs and tissues more closely, neonates were embedded, cryosectioned sagitally, and stained with hematoxylin and eosin (H Figure 2A, F). Compared to age-matched WT littermates, no gross defects in non-neuronal tissue were noted, and all tissues of the nervous system were present and morphologically normal (Figure 2A–J).

**Figure 3:**
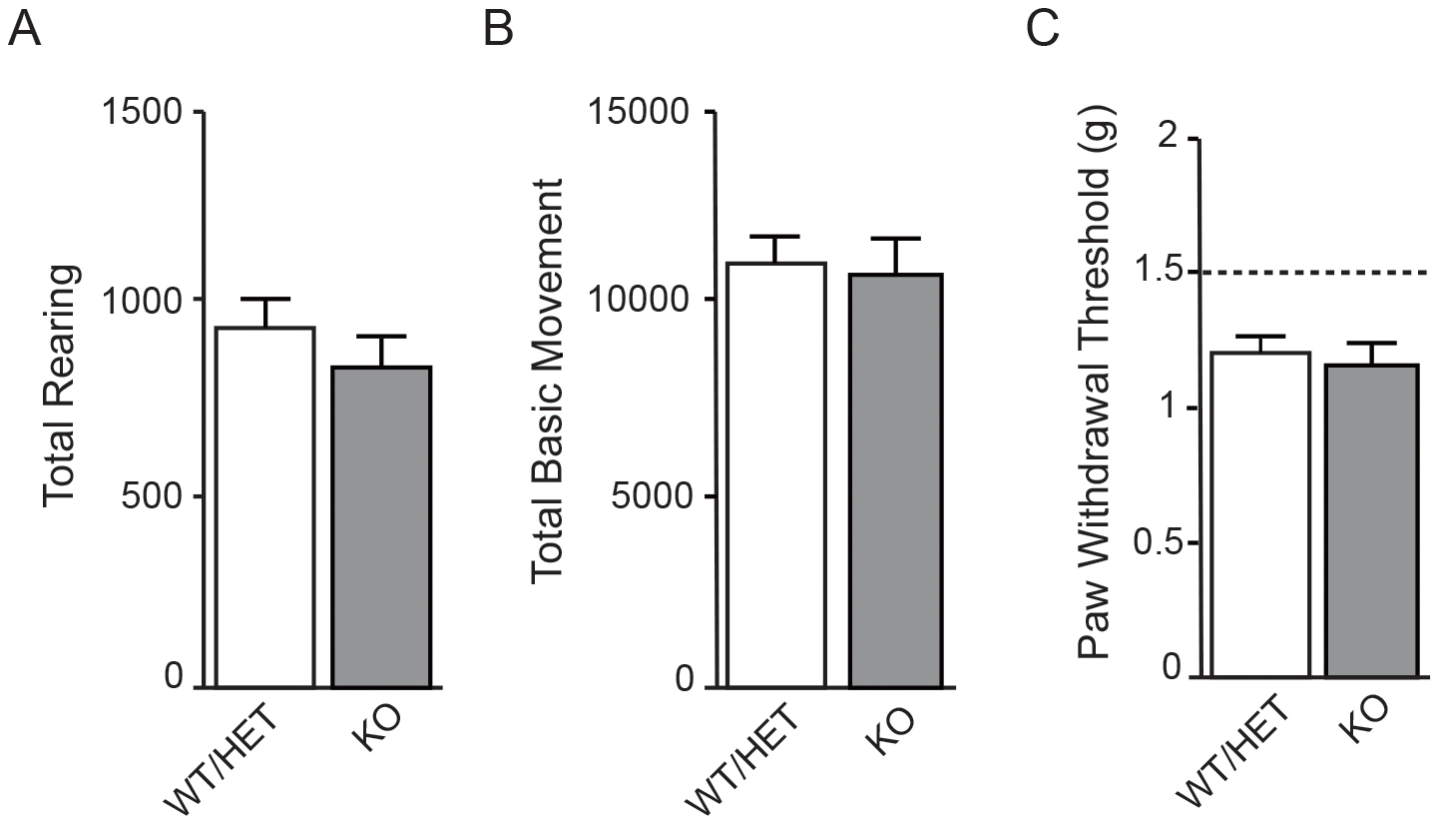
Baseline movement and tactile sensitivity of Nav1.7 KOs are not different from WT/HET. Overall locomotion was evaluated in Nav1.7 WT/HET and KO animals by scoring both total rearing behavior and basic movement using beam breaks in an automated open-field box. **A.** There was no statistically significant difference in rearing behavior between WT/HET (11187±492, n = 20) and KO (11036±781, n = 17) littermates (pairwise t-test, p = 0.2438). **B.** There was no statistically significant difference in basic movement between WT/HET (935±85, n = 20) and KO (835±75, n = 17) littermates (mean ± S.E.M.; pairwise t-test, p = 0.528). **C.** Tactile sensitivity, as assayed by measuring threshold of paw withdrawal to von Frey fibers of increasing force, was not significantly different between WT/HET (1.225 g ±0.05 g, n = 11) and KO littermates (1.18 g ±0.08 g, n = 7) (mean ± S.E.M.; Wilcoxon Two-Sample Exact Test, p = 0.6434). Dashed line represents the level at which the animal’s paw was physically lifted by the von Frey monofilament and is included to show that the measured responses are due to true behavioral tactile response.

Consistent with the normal anatomy, in behavioral testing KOs showed overall movement and tactile sensitivity indistinguishable from that of WT/HET littermates. In open-field testing, total rearing and total basic movement each were identical between KOs and control littermates (Figure 3A-3B). Mechanical sensitivity was similar between KO and WT/HET littermates, as assessed by testing threshold to withdrawal in response to von Frey fibers of varying stiffness (Figure 3C). All these data are consistent with reports from human CIP patients lacking functional Nav1.7, whose only reported defects are in pain sensation and in ability to smell [1]–[3],[20].

### Nav1.7 global knockout mice do not respond to nociceptive stimuli

**Figure 4:**
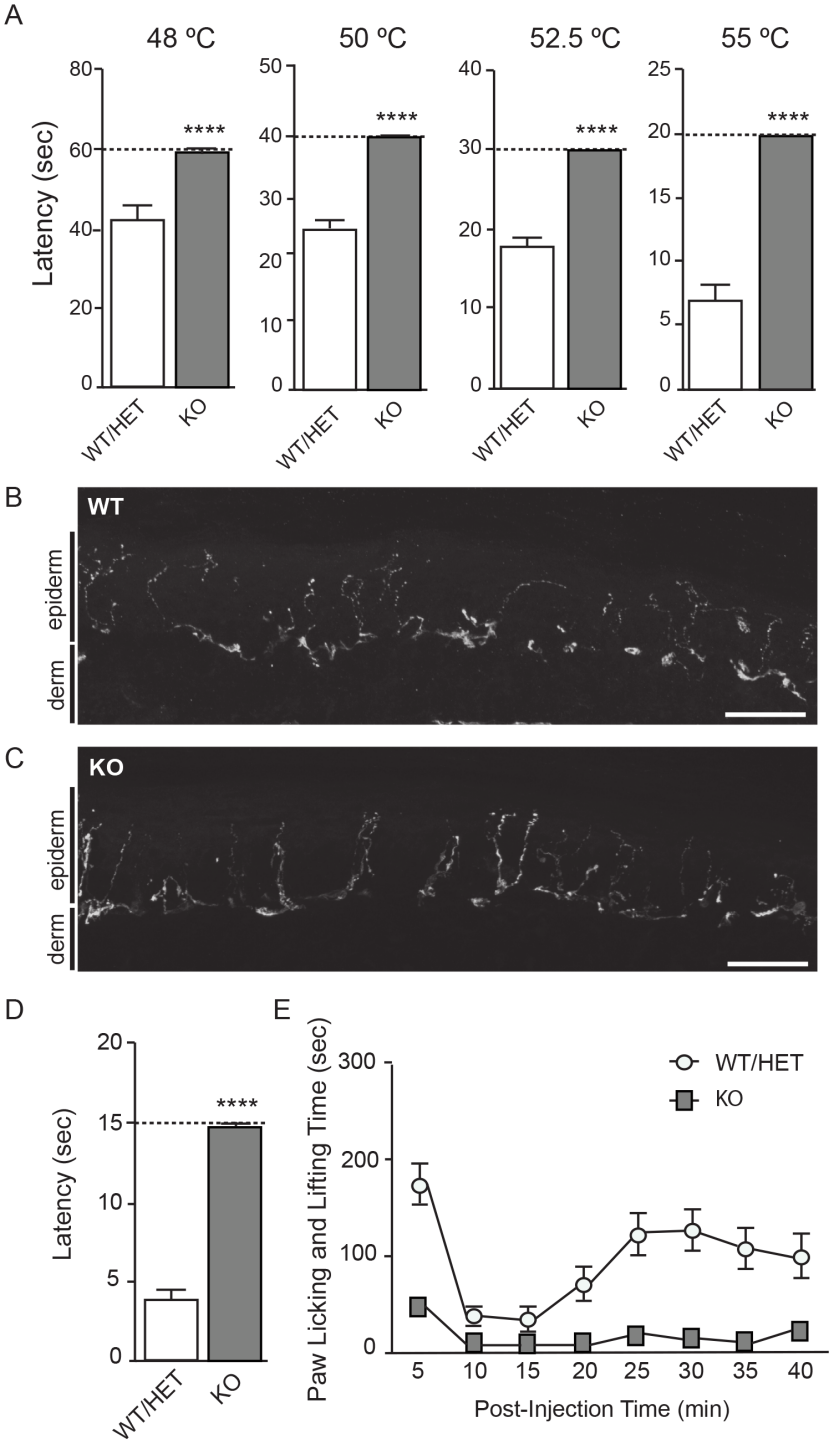
Nav1.7 KOs are insensitive to painful thermal, mechanical, and chemical stimuli. **A.** Latency of paw withdrawal in hot plate testing from Nav1.7 KOs (n = 18) and WT/HET littermates (n = 20). Withdrawal latency was tested at temperatures ranging from 48°C to 55°C as noted atop each panel. For WT/HET animals, withdrawal latency decreased with increasing temperature (note different y-axis scale for each panel). At each temperature tested, KO animals showed no response to thermal insult and were removed from the heat source at a predetermined cutoff time (dashed line) to avoid damage to superficial tissue. In all cases the difference between WT/HET and KO was statistically significant. (**48°C**: WT/HET, 42.2 sec ±2.6 sec and KO, 59 sec ±1 sec; **50°C**: WT/HET 25.2 sec ±1.5 sec and KO, 59 sec ±0.2 sec; **52.5°C**: WT/HET, 17.7 sec ±1.2 sec and KO, 30 sec ±0 sec; **55°C**: WT/HET, 6.9 sec ±0.9 sec and KO, 20 sec ±0 sec (mean ± S.E.M., Wilcoxon two-sample exact test, 48°C: p = 4.470 E-08; 50°C: p = 3.806 E-08; 52.5°C: p = 5.956 E-11; 55°C: p = 5.956 E-11). **B–C.** Immunofluorescence labeling of epidermal nerve fibers from adult Nav1.7 WT (**B**) and KO littermates (**C**) (Scale bars in **B** and **C** = 150 µm). Epiderm and derm border is indicated on the left hand side of each panel. **D.** Latency time to response to noxious mechanical stimulus (tail clip), tested in Nav1.7 KOs (14.8 sec ±0.2 sec, n = 10) and WT/HET littermates (4.1 sec ±0.7 sec, n = 29) (mean ± S.E.M., Wilcoxon two-sample exact test; p = 2.2883 E-08). To prevent tissue damage, clip was removed from animals that did not withdraw by the predetermined time of 15 sec, and the time was scored as 15 sec. **E.** Formalin test on Nav1.7 KO and WT/HET littermates. Graph shows the total time spent in licking and lifting behaviors, binned at five minute intervals, for KO (dark squares, n = 13) and WT/HET (hollow circles, n = 12). Total flinches in phase I (0–10 minutes) were 46.6±8.7 from KO and 173.9±20.9 from WT/HET, and total flinches in phase II (15–40 minutes) were 86.5±19.4 from KO and 603.9±79.7 from WT/HET (mean ± S.E.M., ANOVA, p<0.0001 for both phases). Note that the non-zero baseline of paw licking and lifting behavior also reflects a component of residual non-painful paw licking and lifting that occurs absent formalin stimulus (not shown). No statistically significant difference in paw edema following the experiment was detected between the WT group (1.09 mm ±0.14 mm) compared to the HET (0.71 mm ±0.07 mm) or KO (0.68 mm ±0.06 mm) groups (mean ± S.E.M, ANOVA, p = 0.0624, F_2,22_ = 3.155). Although there was a trend towards more edema in the WT group, the sample size did not lend itself to determining statistical significance.

Pain sensitivity in adult CD1-Nav1.7 KO and BALB/c-Nav1.7 KO mice was quantitated with hot plate, tail clip, formalin, and complete Freund’s adjuvant (CFA) models of pain to test, respectively, sensitivity or sensitivity thresholds to thermal, mechanical, chemical-induced, and inflammation-induced pain. In all behavioral tests the knockouts showed dramatic differences from control littermates, differences so large that we ascribe them to missing Nav1.7. In the hot plate model of pain, withdrawal latency of WT/HET mice decreased as temperature increased, as anticipated, up to a maximum of 55°C (Figure 4A). At each temperature, latency to withdrawal was significantly longer for KO mice than WT/HET. Moreover, KO mice did not display painful behaviors up to the predetermined maximum latency time (Figure 4A, dashed line in each panel) at which mice were removed from the hot plate to prevent tissue damage, suggesting KOs were insensitive to acute thermal pain at these temperatures. Thermal hyperalgesia (Hargreaves test) was assayed as part of the CFA model (see below). Importantly, innervation of the skin, which presumably reflects the architecture necessary for initiation of pain (and other sensory modalities) at the periphery, was not observably different between WT/HET and KOs (Figure 4B–4C respectively). Sensitivity to mechanical pain was tested with a tail-clip assay (Figure 4D). Knockout mice were insensitive to mechanical hyperalgesia in this assay and did not react to a clip placed on the tail within the fifteen second maximum time implemented to avoid tissue damage. Especially given the unchanged tactile sensitivity as measured with von Frey fibers (Figure 3C), most likely the KOs were insensitive not to mechanical sensation, but specifically to this mechanical pain.

Given the very strong expression of Nav1.7 in rodent peripheral nociceptors and reported sparse expression elsewhere [8],[9], the simplest explanation for all these cases of pain is that peripheral nociceptive inputs in the knockouts were inoperative – either action potentials were not triggered or did not reach the spinal cord, through failure of transmission or of presynaptic neurotransmitter release.

KOs also displayed dramatically reduced responses in both phases of the mouse formalin pain assay. Phase I of formalin-induced licking and lifting represents more acute pain, more comparable to hot plate, Hargreaves apparatus, and tail clip assays, whereas phase II represents spontaneous ongoing persistent (debatably chronic) pain. Licking and lifting in phase I of the formalin response was approximately 80% less in KOs than in control animals, and little to no licking and lifting was recorded in phase II (Figure 4E). The remaining phase I signal in the KOs could potentially represent some residual sensitivity to pain, or it could represent movement due to handling and/or non-painful sensation from the volume of the injection stretching the dorsal surface of the hindpaw.

**Figure 5:**
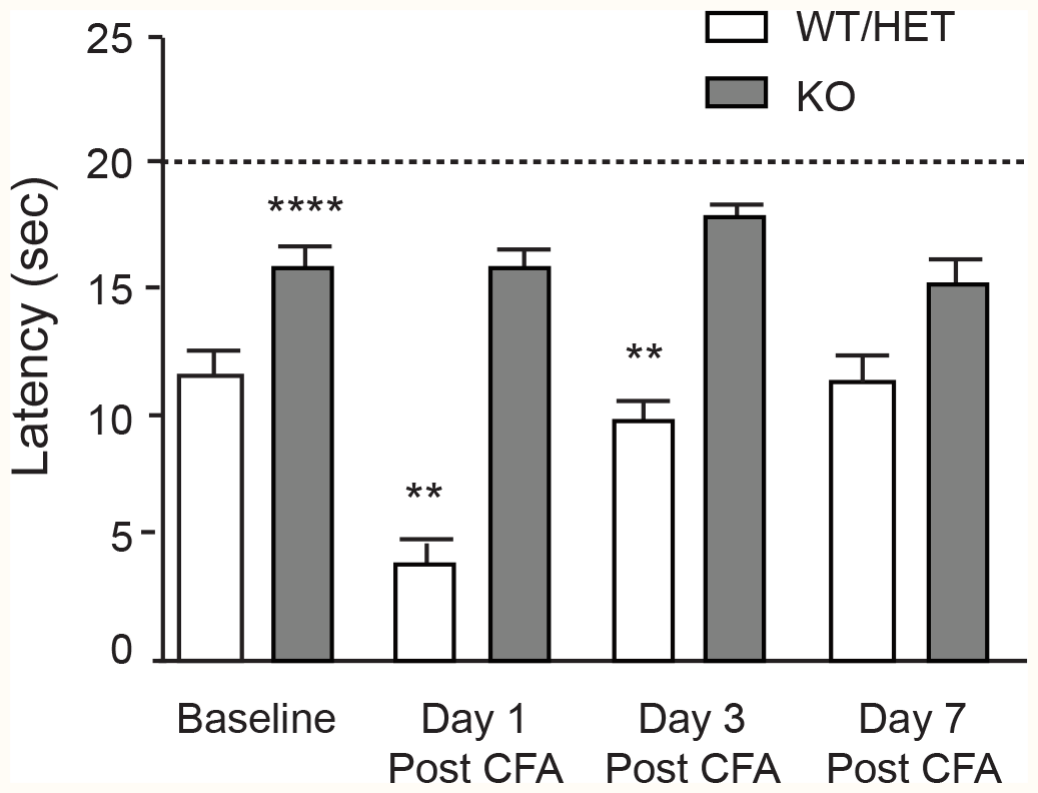
Nav1.7 KOs do not develop CFA-induced thermal hyperalgesia. Latency time to withdrawal from radiant heat stimulus for Nav1.7 KO (n = 4) and WT/HET littermates (n = 9) at baseline and 1, 3, and 7 days following CFA injection. WT/HET mice developed robust hyperalgesia in the affected paw 24 hours post-CFA injection that returned to baseline at day 7. KO mice did not develop hyperalgesia. Differences between KO and WT/HET were statistically significant at baseline (WT/HET = 11.5 sec ±0.8 sec; KO = 16 sec ±0.8 sec, p = 0.0001), day 1 (WT/HET = 4.0 sec ±0.5 sec, KO = 15.7 sec ±0.9 sec, p<0.0001) and day 3 (WT/HET = 9.6 sec ±1.0 sec, KO = 17.8 sec ±0.7 sec, p = 0.0028), but not at day 7 (WT/HET = 11.3 sec ±1.1 sec, KO = 15.3 sec ±1.0 sec, p = 0.5381), at which time control animals had recovered back to baseline (all values mean ± S.E.M, ANOVA).

Persistent and chronic pain by definition involve sensitization or remodeling of the nervous system, and neuropathies and other chronic pain indications represent the main unmet medical need for analgesics [30]. Although it seems clear that Nav1.7 is crucial for nociceptive pain, and clinical genetics indicate aberrant Nav1.7 can play a role in human neuropathies [5], it is unclear to what extent Nav1.7 governs pain following pathological neuronal sensitization. To address one form of this we evaluated Nav1.7 KOs on the Hargreaves apparatus for withdrawal latency to radiant heat stimulus before and after injection of complete Freund’s adjuvant (CFA) into the paw (Figure 5). Before CFA administration (baseline), WT/HET mice reacted at an average of 11.5 seconds, while KOs reacted with a statistically significant increase in latency time, averaging 16 seconds. Even with this approximate 40% increase in latency time, KO mice did still react to the stimulus at less than the cutoff time of twenty seconds. This could reflect some remaining residual pain sensitivity in the KOs, or, as we speculate, reaction to the non-noxious stimulus (e.g., warmth, touch and handling) that necessarily accompanies the noxious painful stimulus, similar to the small response seen in phase I of the formalin assay. Twenty four hours following CFA administration, WT/HET mice had a much shortened latency to withdrawal, showing the induction of thermal hyperalgesia, and this returned to baseline by day 7. Nav1.7 KOs did not develop thermal hyperalgesia at any time point measured, although there was an immediate transient swelling and edema visible at the paw upon CFA injection. The lack of thermal hyperalgesia in the KOs at any time point implies that Nav1.7 governs pain even following sensitization of the nervous system.

### Nav1.7 global knockout mice display reduced TTX-sensitive currents in sensory neurons and reduced C-fiber spiking

**Figure 6:**
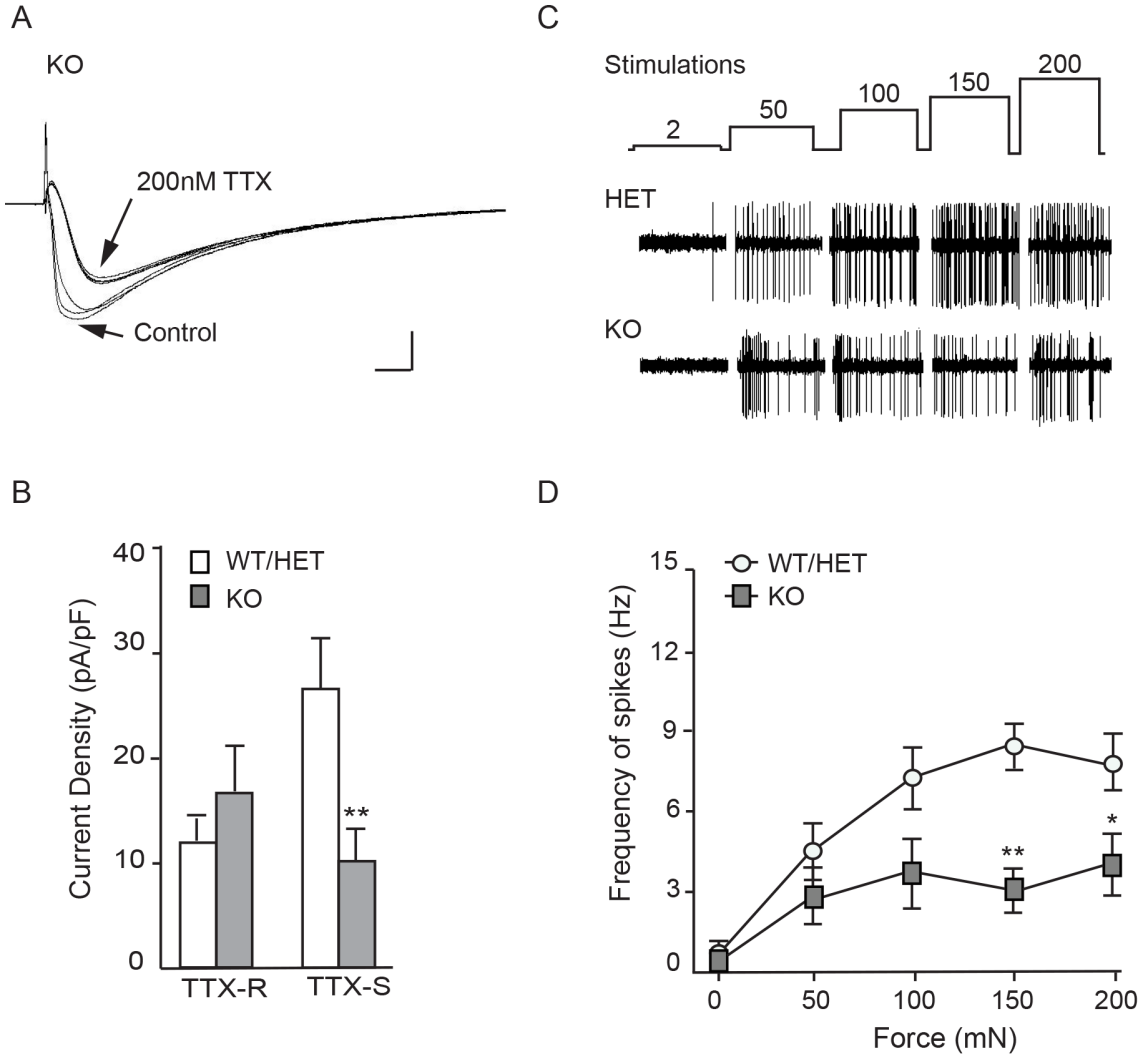
TTX-sensitive currents and mechanically-evoked spiking of C-fibers are reduced in KO mice. **A**. Representative current traces showing the effect of 200 nM TTX on a KO DRG neuron. Overlaid sweeps show the time course of selective TTX blockade of fast-inactivating currents. Sweeps shown are the three before and the three after TTX addition (inter-sweep interval 10 s). Scale bars, 200 pA and 2 ms. **B**. Average current densities from acutely dissociated Nav1.7 KO and age-matched WT adult DRG neurons. TTX-S currents: 10.1±3.06 pA/pF, n = 12 neurons from 2 KO animals; and WT/HET, 27.2±4.53 pA/pF, n = 16 neurons from 4 control animals (2 WT and 2 HET) (p = 0.0074, unpaired t-test, mean ± S.E.M.). TTX-R currents: 16.9±4.44 pA/pF from KO, 11.9±3.26 from WT/HET (p = 0.3638, unpaired t-test, mean ± S.E.M.). Cell capacitance averaged 50.2 pF ±5.7 pF from WT/HET and 65.4 pF ±8.6 pF from KO (p = 0.069, unpaired t-test, mean ± S.E.M.). **C**. Representative mechanically-evoked action potentials in saphenous nerve. Upper trace shows the stimulation protocol (mN); middle and lower traces show representative action potentials from a single C-fiber from a Nav1.7 HET and a Nav1.7 KO preparation respectively. **D.** Frequency of mechanically-evoked action potentials from C fibers was significantly reduced in Nav1.7 KO mice (filled squares, n = 9 fibers) at forces ≥150 mN when compared to those recorded in C fibers from Nav1.7 control animals (open circles, n = 13 fibers) (150 mN: 8.71±0.86 Hz in control; 3.20±0.76 Hz in KO (mean ± S.E.M., two way ANOVA; p<0.01); 200 mN: 8.1±1.11 in control, 4.14±1.14 Hz in KO (mean ± S.E.M., two way ANOVA, p<0.05)).

Whole-cell patch clamp recordings were made from the cell bodies of neurons acutely dissociated from dorsal root ganglia to determine the contribution of Nav1.7 to overall sodium current. As expected, neurons showed a fast-inactivating component sensitive to tetrodotoxin and a slowly-inactivating tetrodotoxin-resistant component [31]–[34] (Figure 6A). Both these currents were present in neurons isolated from WT/HET and in neurons isolated from KO mice, and neurons from both genotypes were found that expressed mostly TTX-S, mostly TTX-R, and mixed sodium current. The overall density of TTX-S current was lower in neurons from KO than from WT/HET, demonstrating that some but not all TTX-S current in mouse DRG neurons is encoded by Nav1.7. The density of TTX-R current was not statistically different (Figure 6B). To investigate the role of Nav1.7 in axonal transmission, spiking was recorded from single C-fibers in saphenous nerve in response to mechanical stimuli using a skin-nerve preparation [35]. Spontaneous action potential frequency was 6-fold lower in KO than in WT/HET mouse preparations. Likewise, mechanical force-response analysis revealed that C-fiber action potential frequency was reduced in KO compared to WT/HET mouse preparations, reaching statistical significance at the higher spiking rates evoked by stronger stimulus (Figure 6C –6D). Results suggest Nav1.7 mediates axonal transmission, but is not exclusively responsible for initiation and propagation in this particular fiber type and preparation. Note that any fiber types completely insensitive to the electrical or mechanical stimuli used to identify fibers for an experiment would not have been included in our analysis.

### Nav1.7 global knockout mice are insensitive to pain resulting from activation of peripheral sodium channels

**Figure 7:**
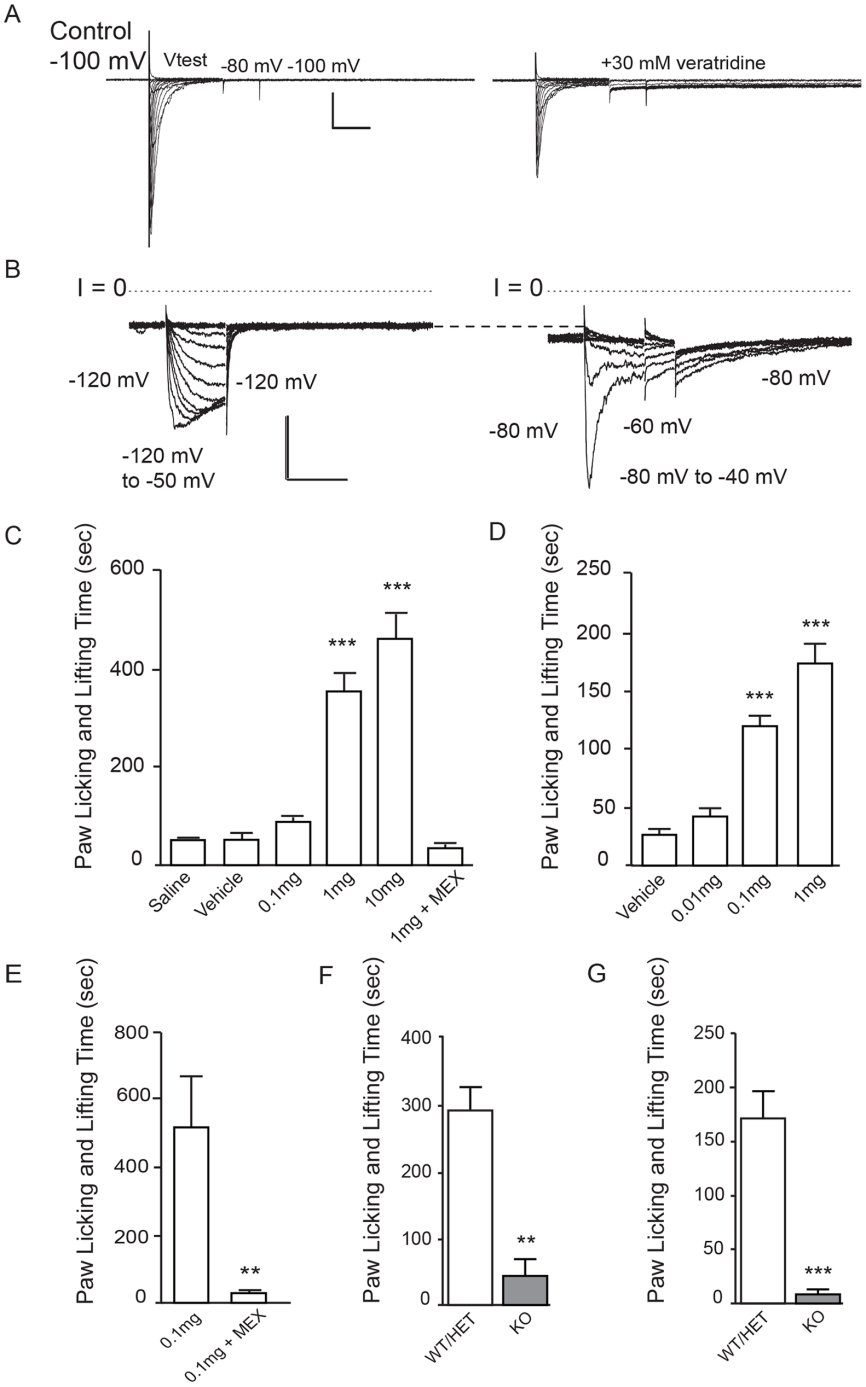
Nav1.7 KOs show minimal pain behaviors upon injection of veratridine or grayanotoxin III. **A.** hNav1.7 currents recorded from HEK 293 cells in control (left) and after addition of 30 µM veratridine (right). Currents were evoked by a family (traces overlaid) of depolarizing voltage pulses incremented by +10 mV from a holding voltage of −100 mV, with repolarization to −80 mV followed by return to the holding voltage of −100 mV. Inward currents were evoked by depolarizations to −50 mV and more positive. Note the prolonged opening following repolarization in veratridine. Scale bars, 500 pA and 10 ms. **B.** Nav1.7 currents recorded with 300 µM grayanotoxin III in the internal (pipette) solution. Currents shown at left are in response to a family (traces overlaid) of step depolarizations in +5 mV increments from −120 mV to −50 mV, followed by repolarization to −120 mV. Depolarizations to −95 mV and more positive evoked inward currents, and these currents showed little or no inactivation during the test pulse. Dotted line marks the zero current level (I = 0). Currents shown at right are records from the same cell, after switching the holding voltage to −80 mV. Note that the holding current increased despite the decreased driving force (compare current at −80 mV to the dashed line marking the holding current at −120 mV), presumably reflecting steady influx of sodium ions through grayanotoxin-modified Nav1.7. Step depolarizations from −80 mV to −40 mV at +10 mV increments evoked additional currents with slow inactivation and deactivation kinetics. Scale bars, 500 pA and 20 ms. **C.** Licking and lifting in male CD-1 mice in response to increasing intraplantar doses of veratridine. At doses of 1 µg and 10 µg, veratridine caused a statistically significant increase in paw licking and lifting behaviors compared to saline or vehicle (1% ethanol/99% PBS) controls. The licking and lifting caused by 1 µg veratridine was completely prevented by pre-dosing the animals with mexiletine (MEX; 30 mg/kg, i.p. or p.o.). A separate animal cohort was used for each dose. **D.** Licking and lifting in male CD-1 mice in response to increasing intraplantar doses of grayanotoxin III. At doses of 0.1 µg and 1 µg, grayanotoxin III caused a statistically significant increase in paw licking and lifting behaviors compared to saline or vehicle (1% ethanol/99% PBS) controls. A separate animal cohort was used for each dose. **E.** In a separate experiment, the licking and lifting induced by grayanotoxin III was prevented by pre-dosing the animals with mexiletine (MEX; 30 mg/kg, i.p. or p.o.). **F.** Total paw licking and lifting behavior time in Nav1.7 KO (n = 5) and WT/HET littermates (n = 7) mice in the 20 minutes following i.pl. injection of 1 µg veratridine. Responses from KO (44.4 sec ±26.6 sec) were smaller than from WT/HET (292.1 sec ±34.7 sec) (p = 0.0073) (mean ± S.E.M., homogeneous ANOVA model with Tukey-Kramer adjusted t-test). **G.** Total painful paw lifting and licking behavior time in Nav1.7 KO (n = 3) and WT/HET littermates (n = 7) in the 15 minutes following i.pl. injection of 0.1 µg grayanotoxin III. Responses from KO (7.33 sec ±3.5 sec) were smaller than from WT/HET (170.9 sec ±23.1 sec) (p = 0.0003) (mean ± S.E.M., heterogeneous ANOVA model with Welch’s test).

As human inherited erythromelalgia (IEM) and paroxysmal extreme pain disorder (PEPD) are caused by mutations that increase Nav1.7 opening [11], we attempted to mimic the effects of such mutations pharmacologically with the lipophilic small molecules veratradine and grayanotoxin III. Tested with patch-clamp electrophysiology on human Nav1.7 expressed in HEK-293 cells, veratradine (Figure 7A) and grayanotoxin III (Figure 7B) each caused channels to remain open upon repolarization following step depolarization [36],[37]. (Lower peak currents in veratridine were likely due to occupancy of a second open state with lower conductance [37].) Channels in the presence of grayanotoxin III were open tonically at voltages corresponding to resting membrane voltage and had altered inactivation kinetics. When injected into the paws of mice, each of these compounds produced a robust licking and lifting response of the affected limb. Grayanotoxin III and veratridine each bind at the batrachotoxin/local anesthetic site common to all sodium channels [38], and insofar as reported, each is nonselective among the sodium channel family and so could be expected to activate all sodium channel subtypes, with similar potency [39]. Toxins each caused a dose-dependent increase in pain-like lifting and licking behaviors, and these behaviors were prevented by systemic pre-administration of the nonselective sodium channel inhibitor mexiletine (Figure 7C–7E). Since two structurally different sodium channel activators produced dose-dependent pain-like behaviors, prevented by pre-administration of a sodium channel antagonist, we conclude that these openers produced pain via direct opening of sodium channels, likely of peripheral nerves. Tested in parallel on KO and WT/HET animals, one microgram veratridine produced lifting and licking response 85% lower in KO mice than in WT/HET mice (Figure 7F). Similarly, 0.1 microgram grayanotoxin III caused a robust painful response in WT/HET mice that was 96% lower in KOs (Figure 7G). In vivo distribution of the toxins is unknown, as is the distribution of different sodium channels, and it is not possible to say what exact voltage stimulus various sodium channels received in vivo. It is possible that following in vivo administration the only sodium channel the agonists contacted or opened was Nav1.7, but this seems somewhat unlikely given the coexpression of multiple sodium channel subtypes in peripheral processes [40] and in sensory ganglia [25], and since innervation of the skin is apparently similar in KO and WT/HET mice (see Figures 3 and 4). Whether toxins triggered noxious stimulus via direct action on Nav1.7, on other sodium channels, or on a combination of channels [41], results again show a critical role for Nav1.7 in the transduction of pain, possibly even compared to other peripheral sodium channels.

### Anosmia and lack of response to histamine in Nav1.7 knockouts

**Figure 8:**
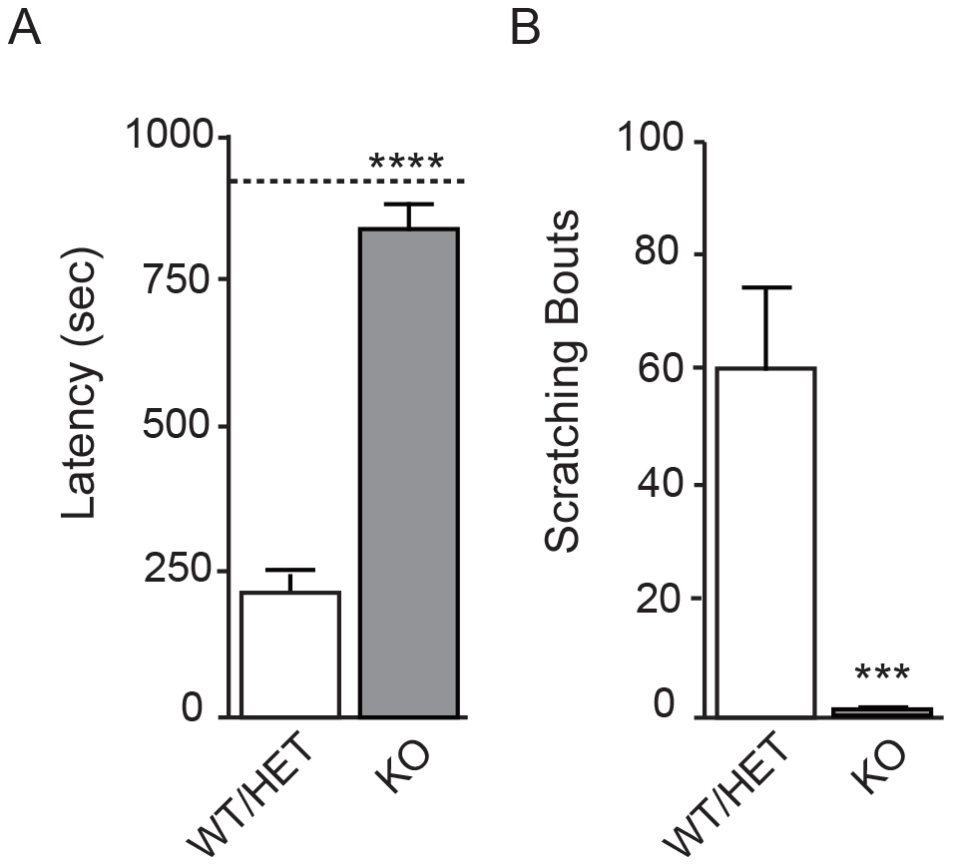
Nav1.7 KOs display inability to smell and insensitivity to intra-dermal histamine. **A.** Total time spent to find a scented food pellet buried in cage bedding for Nav1.7 KO (n = 19) and WT/HET littermates (n = 21). Only one KO found the pellet before the test was cut off at 15 minutes; for statistical purposes the other 18 KO animals were each assigned a time of 15 minutes (900 sec) for an average time of 838.4 sec ±40.2 sec. All WT/HET animals found the pellet, in average time 214.9 sec ±39.3 sec (p = 2.209 E-09) (mean ± S.E.M., Wilcoxon two-sample exact test). **B.** Total number of scratching bouts following i.d. injection of histamine for Nav1.7 KO (n = 8) and WT/HET littermates (n = 17). Nav1.7 KO animals showed a 98% reduction in scratching bouts (average 1.25±0.6) compared to WT/HET (60.9±13.7) (mean ± S.E.M., heterogeneous ANOVA analysis, p<0.0001).

We next evaluated olfaction in Nav1.7 KOs by use of a buried food assay. Mice were habituated to feed on scented pellets and then were fasted for 24 hours. WT/HET mice found the hidden food pellet with a fairly consistent latency time of three and a half minutes (Figure 8A). Of the 19 KOs tested, 18 did not find the pellet before the test was ended at 15 minutes (900 sec). One mouse found the pellet before cutoff, but from behavioral observation this mouse may have found it through random scuffling of the cage bedding (alternatively, this mouse may have had some residual smell sense). Data are consistent with reported behavioral characterization of mice with olfactory-specific Nav1.7 disruption, which were unable to sense a variety of odors [20]. We conclude that global Nav1.7 KO mice are anosmic, consistent with the inability of human Nav1.7 CIP patients to smell [1],[20]. Finally, knockouts and WT/HET mice were subject to histamine challenge. Although intra-dermal histamine injection produced a robust scratching response in WT/HET mice, histamine produced few to no scratching bouts in KO mice (Figure 8B), similar to saline injection (not shown). The scratching response to histamine may reflect pain, itch, or a combination, considering the uncertain correspondence between histamine and itch in rodents. Histamine did produce a classic flare response at the site of injection in the KOs (not shown), suggesting normal immune reaction. Interestingly, recent profiling shows that genotyped human CIP patients respond to histamine challenge with a flare but do not itch (M. Koltzenburg, personal communication), and that gain-of-function variants in Nav1.7 produce itch in humans [42]. The area remains to be explored further, since in early clinical profiles, CIP patients (not genotyped) were observed to scratch spontaneously [43], and at times we observed spontaneous scratching in Nav1.7 KO mice.

## Discussion

Our experiments show conclusively that in mice, as in humans, there is nothing critical about Nav1.7 for survival or for most functions. A driver of the previously reported neonatal lethal phenotype likely was linked to inbred background, since in our hands KOs on the congenic C57Bl6 or BALB/c backgrounds could not be rescued despite all husbandry and caring procedures. Loss of smell resulting in inability to find food almost certainly played a major role in lack of survival [11],[20], but this effect is modified by genetic background, since a small portion of our KO animals on hybrid backgrounds survived even without hand-feeding or other human intervention. The particular alleles involved are unknown. All behavioral abnormalities we noted in the KOs, whether formally quantitated or anecdotally, could plausibly be explained by inability to smell or by inability to sense pain. Heterozygous mice were indistinguishable from homozygous wildtype mice, in informal observation and in the quantified behavioral profiling undertaken. Hybrid generation through back-crossing or out-crossing plus husbandry as described here may be helpful in creating global knockouts of other genes that on first look give an apparent neonatal-lethal phenotype.

Insofar as tested, the global knockout mouse recapitulates the human CIP phenotype [1]. The most striking feature of the KO mice is the apparent absence of pain in response to validated thermal (hot plate, radiant heat), mechanical (tail clip), and chemical (formalin, sodium channel agonists) challenges, and in response to persistent painful inflammatory sensitization (CFA treatment). These tests are not comprehensive of all pain insults and models [22], but together the behavioral endpoints of the models tested are thought to encompass peripheral input, spinal cord excitability, descending inhibitory pathways, and peripheral and central sensitization [44],[45]. Nav1.7 may operate within each of these pathways, or possibly could govern pain endpoints of these assays entirely via peripheral nociceptive input to the spinal cord. The strength of the behavioral phenotype of the Nav1.7 KO is notable: many mutant mice with reduced pain phenotype have been reported, but none that have complete abolition of pain phenotype without additional neurological effects that would confound a pain readout [46]. It is interesting that whereas Nav1.7 KOs had complete loss of pain, significant sodium current still remained in the cell bodies of sensory neurons, and axonal spiking frequency of those C-fibers measured was reduced but not eliminated. This suggests either that an incomplete reduction in peripheral excitability produces complete shutoff of pain, or, possibly more likely, that the critical cell types or subcellular areas at which Nav1.7 controls pain were not recorded in these preparations. We speculate that the key pain pathway controlled by Nav1.7 is the transmission of action potentials along axons of peripheral nociceptive neurons and propagation through fine nerve endings into the spinal cord [9],[20],[47]. This would be analogous to the olfactory system, in which synaptic transmission from olfactory glomerular cells onto olfactory relay neurons is lost wholly upon deletion of Nav1.7 [20]. Loss of Nav1.7 either at peripheral axons or at fine nerve terminals within the spinal cord could produce shutoff of peripheral nociceptive signals, consistent with analgesic effects of an inhibitory anti-Nav1.7 antibody [48]. Nav1.7 may also govern pain within the CNS or via excitability of sympathetic neurons [21],[22],[30],[49], but sympathetic neurons may be more likely to modulate pain sensation than govern it entirely. More complex scenarios are possible.

Much more complex pain-related phenotypes than those presented here have been reported from transgenic mice expressing floxed Nav1.7 alleles and subject to promoter-specific removal [19],[21],[22]. Some clear differences to our results are seen, such as the unchanged response in the hot plate test of mice in which Nav1.7 has been conditionally removed from all Nav1.8-expressing nociceptors (Nav1.8 Cre approach) or from all sensory neurons (Advilin CRE approach). On the other hand, deleting Nav1.7 more broadly (Advilin/Wnt1 CRE approach) recapitulated in hot plate the pain-free phenotype seen in global KO mice and in CIP patients, but did not affect sense of smell. In general, comparison of the amount and the tissue distribution of promoter-specific Nav1.7 deletion is necessary to compare specific perturbations to the global Nav1.7 knockout presented here. The different approaches are complementary, as global knockouts themselves come with the caution that compensatory regulation of other genes is possible, as the tradeoff for unambiguous deletion of the gene in all tissues [50]. Many sodium channels indeed have demonstrably plastic expression upon injury or insult [51] and we do not rule out compensatory changes in expression of other sodium channels. The normal movement and tactile sensitivity of the knockouts, however, and the similar amount of TTX-R current recorded from cell bodies of DRG neurons suggest no drastic decreases in the expression of other sodium channels. Conversely, any sodium channels that were up-regulated were unable to compensate for the role of Nav1.7 in mediating pain.

The effect of global Nav1.7 deletion is much stronger than the effects of global deletion of other peripheral sodium channels. Global knockouts of Nav1.3, Nav1.8, and Nav1.9 [23],[52]–[54] show comparatively subtle phenotypes in pain assays. There generally are no data on the pain sensitivity of humans or animals missing sodium channels with major CNS, cardiac, or skeletal muscle expression; the phenotypes generally are too severe for meaningful pain testing [55],[56]. The insensitivity of the Nav1.7 KOs to pain induced by nonselective sodium channel activators suggests that opening of a peripheral non-Nav1.7 sodium channel either does not trigger nociceptor spiking, or that propagation of the action potential into the spinal cord still depends entirely on Nav1.7 function. Absent direct functional measurements of veratridine and grayanotoxin III on each sodium channel subtype, however, plus in vivo distribution of the toxins and knowledge of the in vivo voltage stimulus, this cannot be stated definitively.

From a drug discovery perspective, the phenotype of the KO provides guideposts to the development of Nav1.7-selective sodium channel inhibitors, a class of compounds under active pursuit [57]. A global knockout represents the extreme of on-target blockade, and the normal anatomy and movement of the KO imply no safety-related on-target toxicity to a Nav1.7 inhibitor. Accordingly, future preclinical toxicology findings with Nav1.7 inhibitors may at first pass be attributed to off-target effects. We speculate that a state-dependent pharmacological Nav1.7 inhibitor might inhibit firing of olfactory neurons less than the abnormally firing neurons encoding pathological pain, and thereby avoid even this potential on-target liability, but this remains to be tested. Work here also shows that multiple pain and possibly itch models could be appropriate preclinical and in some cases possibly translatable driver models for testing efficacy and doses of specific Nav1.7 inhibitors. The relation between target occupancy and behavioral outcome might be different for each model, however, since small molecule sodium channel inhibitors tend to have strongest potency with stimuli that promote channel inactivation [57]–[59], and neuronal firing likely is different for each model. It is difficult to extrapolate from preclinical pain models to a particular human clinical pain syndrome that is most likely to be treatable by inhibition of Nav1.7. No cases of neuropathic pain in CIP patients are reported, but the number of patients likely is too small to draw a conclusion about whether, for example, painful diabetic neuropathy or HIV-dependent neuropathy, two of the largest populations of neuropathic pain, can develop without functional Nav1.7.

Our results to date suggest a comparatively simple view of the role of Nav1.7. In mice as in humans, it governs acute pain, olfaction, and little else. Other mechanisms and other sodium channels would of course govern signaling in spinal cord and brain areas of the pain matrix where peripheral signals actually become pain [60]. It is tempting to speculate that the emergence of SCN9A in tetrapods via duplication of the SCN1A ancestor [61] enabled the separation of pain from other sensory modalities, and that SCN9A orthologs could govern similar functions in mammals, reptiles, and birds. For teleosts and other non-tetrapod species, then, pain might be a much less distinct sensory modality.

## Materials and Methods

### Nav1.7 mouse colony

All mice used in these studies were cared for in accordance to the Guide for the Care and Use of Laboratory Animals, 8th Edition. Animals were singly housed at weaning age at an AAALAC, internationally accredited facility in sterile ventilated micro-isolator housing on diamond soft bedding. All research protocols were approved by Amgen Inc. Institutional Animal Care and Use Committee (IACUC), Cambridge, MA. Animals had *ad libitum* access to pelleted feed (Harlan Laboratories, South Easton, MA) and reverse osmosis-purified water (Edstrom Industries, Waterford, WI) via automatic watering system. Animals were maintained on a 12∶12 hour light:dark cycle in rooms at temperatures ranging between 20°C to 23.3°C and humidity of 30% to 70%, and had access to enrichment opportunities. All animals were determined specific pathogen free for all respiratory and enteric bacterial pathogens. All animals were ear punched for identification and genotyping purposes. DNA was extracted using standard genomic DNA extraction kits on an automated QIAcube robot (QIAGEN, Valencia, CA). The following primers (IDT DNA, Coralville, IA), originally designed by Deltagen, were used to genotype all animals: GS (E) 5′ AGA CTC TGC GTG CTG CTG GCA AAA AC 3′; GS (T, E) 5′ CGT GGA AAG ACC TTT GTC CCA CCT G 3′ and Neo (T) 5′ GGG CCA GCT CAT TCC TCC CAC TCA T 3′ under a standard 6 step PCR program. DNA denaturing was performed at 95°C for 7 minutes and 96°C for 10 seconds; annealing at 60°C for 30 seconds, extension at 72°C for 1.5 minute for a total of 35 cycles in addition to a final extension step at 72°C for 7 minutes. The resulting endogenous PCR product was obtained by combining primers GS (E) and GS (E,T) to obtain a DNA fragment of 267 base pairs (bp) and that of the targeted product by combining primers Neo(T) and GS (E,T) to obtain a fragment of 389 bp.

Colony was generated as described in the body of the manuscript. In all behavioral tests, homozygous wild type (WT) and heterozygous (HET) mice were compared separately, and in all cases no statistical distinction was found between these two groups (see Statistical Analysis section below).

### Immunohistochemistry

All animals used for histology purposes were trans-cardiacally perfused using ice-cold phosphate-buffer saline (PBS) (Life Technologies, Grand Island, NY) followed by ice-cold fresh 4% paraformaldehyde/PBS (EMS, Hatfield, PA), until the liver cleared of all signs of blood. Hind paws of Nav1.7 WT and KO adult animals were post-fixed in Zamboni’s fixative overnight and cryoprotected in 30% sucrose/PBS for at least 24 hours. Hindpaw skin was dissected, embedded in Tissue Freezing Media (Thermo Fisher Scientific, Pittsburg, PA), placed in a −20°C freezer to set, and stored therein until ready to use. Tissue blocks were then sliced into 25 µm sections on a Leica CM1850 cryostat (Leica Biosystems, Buffalo Grove, IL), placed onto glass microscope slides (Ink Jet Plus, Thermo Fisher Scientific, Pittsburg, PA), and air dried for 30 minutes at room temperature. All immunofluorescence incubations were performed at room temperature, unless otherwise noted. Slides were acclimated to room temperature for 30 minutes, rehydrated in PBS for 30 minutes, and then blocked for 1 hour in wash buffer: 1M PBS/5% normal donkey serum/0.3% Triton X-100. Intraepidermal nerve fibers were labeled overnight at 4°C using a pan neuronal rabbit polyclonal antibody directed against PGP9.5 (aBD Serotec; used at 1∶750 in wash buffer). Sections were then washed 3 times in wash buffer, one hour each wash, and incubated overnight at 4°C in secondary antibody, donkey-anti-rabbit AlexaFluor-488 (Invitrogen, Carlsbad, CA), diluted 1∶1000 in wash buffer. Finally, tissue was washed 3 times in PBS, mounted using VECTASHIELD hard set media containing dapi (Vector Laboratories Inc., Burlingame, CA), and imaged on an inverted Zeiss LSM 5 Pascal confocal microscope (Carl Zeiss Inc., Thornwood, NY). Images were captured with Zeiss Pascal software version 3.5 SP1.1 and assembled in Image J version 1.46r (32 bit).

### Electrophysiology

#### Heterologous cells

HEK293 cells stably expressing hNav1.7 (Millipore, Cambridge, UK) were voltage-clamped using the whole cell patch clamp configuration. Cells were plated onto 35 mm culture dishes 0 - 24 hours before recording, and tissue culture medium was exchanged for external solution containing (in mM) 140 NaCl, 5 KCl, 2 CaCl_2_, 1 MgCl_2_, 10 HEPES, 11 glucose, pH 7.4 with NaOH. Patch pipettes were pulled from borosilicate glass capillaries (1.2 mm outer diameter, World Precision Instruments) with a P-97 pipette puller (Sutter Instruments) and backfilled with internal solution containing (in mM) 62.5 CsCl, 75 CsF, 2.5 MgCl_2_, 5 EGTA, 10 HEPES, pH 7.25 with CsOH. Pipette resistances were between 1.5 and 2.0 MΩ. After formation of the whole-cell configuration, cells were lifted off the dish bottom with the patch pipette and positioned directly in front of a microarray of glass tubes (each tube internal diameter ∼ 1 mm) with each tube containing continuously flowing control or test solution. A full current-voltage relation was recorded from each cell, and cells with poor space clamp as indicated by discontinuous I-V relation or by prolonged or nonexponential current kinetics were discarded. Solution switching was accomplished by moving the microarray with computer control via an RSC-160 rapid solution exchanger (Bio-Logic, Claix, France). Currents were recorded with an Axopatch 200B patch-clamp amplifier driven by pCLAMP software, filtered (4-pole Bessel) at 5 kHz during acquisition, and digitized at 20 kHz using pClamp9.2. Whole cell capacitance and series resistance were uncompensated, and voltages were uncorrected for liquid junction potentials. Currents shown were not leak subtracted. Recordings were made at room temperature.

#### Acute dissociation of neurons from dorsal root ganglia (DRG)

Adult male and female mice (5–6 months old) were euthanized with CO_2_ (according to IACUC guidelines). DRGs from only the lumbar spine levels were removed bilaterally and placed in Ca^2+^/Mg^2+^-free HBSS (Gibco). DRGs were then transferred to a buffered solution containing L-cysteine (Invitrogen) and papain (3 mg/mL, Worthington) and incubated for 30 minutes in a 37°C incubator. Partially dissociated DRGs were subsequently placed in a buffered solution containing dispase and collagenase (3 mg/mL, Promega) for one hour at 37°C. Following these incubations, single-cell dissociation was achieved by passing digested ganglia ∼30 times through flame-polished Pasteur pipettes of decreasing diameter. The cells were resuspended in DMEM/Hams-F12 medium containing 10% fetal bovine serum/20 mM glutamine (Gibco, Invitrogen) and subsequently plated onto Poly-D-Lysine-coated glass coverslips (Cole-Parmer, Vernon Hills, IL). Neurons were used for recordings following two hours of recovery. Electrodes were pulled from borosilicate glass micropipettes (Sutter Instruments, Novato, CA) with a Flaming Brown P97 micropipette puller and had a resistance of 3–4 MΩ. External solution contained (in mM) 20 NaCl, 5 KCl, 1 MgCl_2_, 10 HEPES, 10 Glucose, 160 mannitol, 0.2 CdCl_2_, pH 7.4 (NaOH). CdCl_2_ was used to block Ca^2+^ currents. Internal solution contained (in mM) 130 CsCH_3_SO_3_, 10 NaCl, 0.5 CaCl_2_, 5 EGTA, 10 HEPES, 3 Mg-ATP, pH 7.25 (CsOH). Solution was applied to the recording chamber via gravity flow from a multi-valve perfusion system (Warner Instruments, Hamden, CT) and removed by vacuum suction. Solution exchange was performed by manually switching between the pinch valves. Onset of data acquisition was within 1 min after establishing whole-cell configuration. Errors were minimized with whole cell compensation, and voltages were uncorrected for liquid junction potentials. Currents shown were not leak subtracted. Voltage-clamped currents were recorded on an Axopatch 200B amplifier (Molecular Devices, Sunnyvale, CA) and stored on a personal computer via a Digidata 1332a analog-to-digital converter (Molecular Devices) at an acquisition rate of 100 kHz. A full current-voltage relation was recorded from each cell, and cells with poor space clamp as indicated by prolonged or nonexponential current kinetics were discarded. All data are presented as means±SEM. As a measure of total tetrodotoxin-sensitive (TTX-S) current we used a subtraction protocol whereby TTX-S peaks were obtained via subtraction of an average of three traces upon TTX addition. Capacitance was calculated from the capacitance transient following a voltage step before compensation at the start of recording from each cell that evoked passive currents only. Excel and Clampfit were used for data analysis. Recordings were obtained from DRGs collected from 2 Nav1.7 KO mice and compared to pooled data from 2 HET and 2 WT mice. All recordings and analysis were performed blinded to genotype.

#### Skin nerve preparation

Mice were euthanized via cervical dislocation under Na^+^-pentobarbital anesthesia (150 mg/kg; i.p.). Methods for isolating and recording from the saphenous nerve-skin preparation were modified from those previously described [35]. Briefly, the saphenous nerve and skin from the medial dorsum of the hindpaw were rapidly dissected, mounted in a custom made organ bath chamber with the corium side up, and bath superfused at speed 15 ml/min with 32°C and oxygen-saturated synthetic interstitial fluid containing the following (in mM): 123 NaCl, 4 KCl, 0.7 MgSO_4_, 1 NaH_2_PO_4_, 2.0 CaCl_2_, 9.5 sodium gluconate, 10 glucose, 7.5 sucrose, and 10 HEPES, osmolarity 295 mOsm, pH 7.4 adjusted with NaOH. The proximal end of the saphenous nerve was passed through a hole into a separate, mineral-oil-filled recording chamber, desheathed, and teased into single fiber for extracellular recordings. Single fiber receptive fields in skin were detected by light pressure with a blunt glass rod. Fiber type was determined by action potential conductance velocity following receptive field electrical stimulation with square wave pulses (0.3–0.5 ms; 4–6 mA) delivered through a stainless steel microelectrode. Fibers with a conduction velocity less than 1.2 m/s were classified as C-fibers. Mechanical responses were evoked by square waves of force using a mechanical stimulator (Dual-Mode Lever Systems, Aurora Scientific Inc., Canada), in ascending order of 2, 50, 100, 150 and 200 mN (10 seconds per force with 200 second inter-stimulus intervals). Signals were recorded by a Neurolog system (Digitimer, UK) and Spike 2 software (Cambridge Electronic Design Ltd, UK) for off-line analysis. Action potentials were discriminated and counted using spike histogram software in Spike 2. For fibers that exhibited spontaneous firing, a baseline firing rate was measured before mechanical stimulation. Stimulus-evoked action potentials were determined by subtracting the baseline firing rate from the firing rate that occurred during mechanical challenge. Recordings were obtained from four Nav1.7 KO mice and compared to pooled data from 3 HET and 1 WT mice. All recordings and analysis were performed blinded to genotype.

### Open field assessment

Locomotor activity was measured using photocells after the animal was placed into a novel open field (Photobeam Activity System, Kinder Scientific, Poway, CA) in lights off conditions. Total rearing and total basic movement were collected automatically over a period of 1 hour.

### Histamine challenge

Mice were habituated to Plexiglas chambers (dimensions: 15.25 cm diameter, 30.5 cm tall cylinder) for at least 30 minutes prior to testing. Mice were briefly removed from their cylinder, restrained by hand and injected intradermally (i.d.) at a site in between the shoulder blades with 150 µg of histamine disphosphate in 100 µl of PBS, and immediately returned. Video cameras located above the chambers recorded activity for 40 minutes post-injection. Digital video files were later scored for total scratching bouts at the site of injection.

### Non-noxious tactile stimulation

Paw-withdrawal threshold (PWT) measures were performed on mice with von Frey monofilaments (Stoelting Company, Wood Dale, IL). Mice were placed in Plexiglas chambers (dimensions: 10 cm diameter, 14 cm tall cylinder with lid) atop wire mesh at least 1 hour before testing commenced. Calibrated monofilaments (range 0.16 g to 2 g) stimulated the intraplantar surface of the left hind paw for a maximum of 6 seconds. The PWT was calculated using the Dixon “up-down” method [62].

### Olfaction

The buried food test was performed on mice as described [63]. The latency to retrieve a buried food pellet (pina colada supreme mini treat, Bio-Serv, Frenchtown, NJ) was measured in fasted mice with a maximum retrieval time of 15 minutes after the initiation of testing.

### Nociceptive Assays

#### Thermal hyperalgesia in Complete Freund’s Adjuvant (CFA)-treated mice

Thermal hyperalgesia was measured on the left hind paw of CFA-treated mice with the Hargreaves apparatus (San Diego Instruments, San Diego, CA). Mice were restrained by hand and injected with 20 µL CFA into the intraplantar surface of the left hind paw. Latency to withdrawal from the thermal stimulus was conducted 24 hours prior to CFA injection and at 1, 3, and 7 days post-CFA injection. For each test session, mice were placed in Plexiglas chambers (dimensions: 10 cm diameter, 14 cm tall cylinder with lid) at least 60 minutes before testing commenced. Thermal latency was recorded on the left hind paw at least 3 times each test session for an overall mean latency measurement.

#### Thermal sensitivity

Mice were placed on a hot plate device (Columbus Instruments). The mean latency to jump, lift, and/or lick a hind paw was recorded at 48, 50, 52.5, and 55 C in three separate trials at each temperature.

#### Noxious mechanical hyperalgesia

All mice were tested using a modification of the method of Takagi [64] with a bulldog clamp calibrated to 700 g using a force gauge based on tail diameter. The clamp was applied to an unrestrained mouse’s tail with a maximum testing time of 15 seconds to avoid tissue damage. The latency to bite, vocalize, or bring the nose within 1 centimeter of the clamp site was recorded.

#### Chemical challenge models

Veratridine, grayanotoxin III, and 2% formalin were utilized for the chemical challenge assays. Mice were placed in Plexiglas chambers (dimensions: 10 cm diameter, 14 cm tall cylinder with lid) atop a glass floor for at least 30 minutes before testing commenced. Mice were briefly removed from their cylinder, restrained by hand, and injected with the appropriate chemical for the challenge. For the formalin model, the dorsal left hind paw was injected with 20 µL of a 2% formalin solution in phosphate buffered saline (PBS). For the veratridine or grayanotoxin III models, the intraplantar left hind paw was injected with 20 µL of veratridine or grayanotoxin III dissolved in 1% ethanol and 99% PBS. After the injection, video cameras located underneath the glass floor recorded activity for 20 minutes for all assays except for formalin, which was recorded for 40 minutes. For the formalin assay, acute phase was defined as 0 to 5 minutes after injection and the late tonic phase as 6 to 40 minutes after injection. Paw edema was measured after the formalin experiment with calipers on the dorsal to ventral plane with the use of a digital caliper, left paw diameter minus right paw diameter. Width of both paws was determined by closing the caliper until it just touched the skin. Digital video files were later scored in 5 minute intervals for total time spent lifting and licking the left hind paw.

### Statistical analysis

Statistical analyses were performed in a Windows VISTA environment using R, JMP, and SAS statistical software packages. Initial exploratory data analysis was completed for each experiment. Boxplots were plotted and potential outliers were identified using Tukey’s method. If necessary, a log or square root transformation was applied to stabilize the variance of the data. Initially for each experiment, the data were analyzed using the three category genotype (HET, WT, and KO). If normal assumptions held, a standard one-way ANOVA was applied. If the data exhibited non-normal behavior (e.g. threshold), the non-parametric Kruskal-Wallis test and pair-wise Wilcoxon Exact tests were used in place of the standard ANOVA. For behavioral analysis, KOs were often distinguishable from WT/HET by somewhat smaller size and by large behavioral differences in most assays. Accordingly, we did not consider it possible to run fully randomized and blinded studies. In all tests, results from homozygous (WT) wildtype and heterozygous (HET) mice were compared separately, and in all cases no statistical distinction was found between these two groups. The decision to combine HET and WT categories was based upon similar coefficient values, an insignificant pair-wise HET vs WT test, as well as visual confirmation from the plotted raw data values. If results indicated combining HET and WT was reasonable, homogeneous and heterogeneous ANOVAs were completed for the two category genotype variable (HET/WT and KO). The best model was chosen based on the lowest Akaike information criterion for finite sample sizes (AICC). The corresponding pair-wise t-test was reported as the final result. The residuals were tested to confirm model assumptions. If the data exhibited non-normal behavior (e.g. threshold), the non-parametric Kruskal-Wallis test and pair-wise Wilcoxon Exact tests were used in place of ANOVA. Specific variations were the inclusion of a positive control (HET/Mexiletine and WT/Mexiletine) for the veratridine study and the application of repeated measures ANOVA with time*genotype interaction for the CFA study. Tukey-Kramer adjustment for multiple testing was applied when appropriate. When multiple strains were present, the effect of strain was tested by including a genotype*strain interaction term in the model. A significant effect indicated that differences in mouse behavior between genotypes varied depending on strain as well.
